# Primary Hyperparathyroidism Due to an Ectopic Parathyroid Adenoma Located in the Posterior Part of the Esophagus

**DOI:** 10.7759/cureus.86572

**Published:** 2025-06-23

**Authors:** Erina Nakao, Tomohiko Kimura, Hideyuki Iwamoto, Fuminori Tatsumi, Hideaki Kaneto

**Affiliations:** 1 Department of Diabetes, Endocrinology and Metabolism, Kawasaki Medical School, Kurashiki, JPN

**Keywords:** ectopic parathyroid adenoma, mibi scintigraphy, parathyroid adenoma, primary hyperparathyroidism, pth

## Abstract

Parathyroid glands typically exist in pairs on the posterior surface of the thyroid, and primary hyperparathyroidism (PHPT) is often caused by adenomas in these locations. However, ectopic parathyroid glands may occasionally develop in unusual sites. We present a case of a 71-year-old female patient who exhibited gradual increases in both serum calcium and parathyroid hormone (PTH) levels, along with the development of osteoporosis, suggesting the presence of PHPT. Routine examination, including ultrasonography, sometimes fails to identify an ectopic parathyroid adenoma. In this case, further investigation using methoxyisobutylisonitrile (MIBI) scintigraphy and contrast-enhanced CT revealed an ectopic parathyroid adenoma located in the posterior part of the esophagus. After that, left parathyroidectomy was performed. Left parathyroid weight was 610 mg, and its size was 23 × 10 × 4 mm. Pathological examination confirmed the diagnosis of ectopic parathyroid adenoma. After the surgery, serum PTH and calcium levels were normalized. This case underscores the importance of considering ectopic parathyroid adenomas when ultrasonography fails to identify the lesion and highlights the importance of MIBI scintigraphy in such cases.

## Introduction

Primary hyperparathyroidism (PHPT) is commonly caused by adenomas (approximately 80%), hyperplasia (about 10%), and, less frequently, carcinomas (2-3%), although there is significant variation based on ethnicity [1,2]. The parathyroid glands are typically located in pairs on the posterior surface of the thyroid; however, they may occasionally develop ectopically due to embryological variations. Ectopic parathyroid adenomas account for approximately 7% of cases, with 2% of these being found in the mediastinum. Notably, the inferior parathyroid glands descend along with the thymus, making them prone to a wide range of ectopic locations, particularly in the anterior mediastinum. Ectopic locations for the superior parathyroid glands include the posterior aspect of the esophagus (13%), the space between the thyroid and trachea (2%), within the thyroid itself (3%), and posterior to the thyroid cartilage (1%). For the inferior parathyroid glands, reported ectopic locations include the cervical thymus (27.5%), para-tracheal or para-esophageal areas (12.5%), and the mediastinal thymus (5%) [3]. In this report, we present a case of PHPT caused by an ectopic parathyroid adenoma located in the posterior part of the esophagus, accompanied by a brief review of relevant literature.

## Case presentation

A 71-year-old female patient with a history of kidney stones, diagnosed 20 years before, presented with elevated serum calcium levels. Four years later, her serum calcium was found to be 11.5 mg/dL, and her whole parathyroid hormone (PTH) level was 22.5 pg/mL, which was mildly elevated, however, suggestive of PHPT. These findings suggest the possibility of PHPT. However, ultrasonography failed to identify an enlarged parathyroid gland, and the patient declined further diagnostic investigations, opting for a period of observation. Over the subsequent years, both serum calcium and whole PTH levels gradually increased. One year prior to her visit, she was diagnosed with osteoporosis (T score -4.1, young adult mean (YAM): 51%, which corresponds to 51% of the YAM bone mineral density commonly used in Japanese practice; Z-score was -1.69) and subsequently sought further evaluation. She was admitted to our department at 71 years old. Her height and body weight were 149.2 cm and 44.6 kg, respectively. Her vital signs were as follows: temperature, 36.4°C; blood pressure, 135/54 mmHg; and heart rate, 88 beats/minute. Table 1 shows clinical data for this subject on admission. Laboratory results were as follows: serum calcium, 12.9 mg/dL; phosphate, 2.2 mg/dL; albumin, 4.6 g/dL; whole PTH, 87.4 pg/mL; intact PTH, 172 pg/mL; PTH-related peptide (PTHrP), <1.0 pmol/L; calcitonin, <0.5 pg/mL; renal cyclic adenosine monophosphate (cAMP), 284 nmol/dL/GF; 25-hydroxyvitamin D3 (25(OH)D3), 16.1 μg/dL; 1,25-dihydroxyvitamin D3 (1,25(OH)D3), 72 pg/dL.

**Table 1:** Clinical data in this subject on admission ALT, alanine aminotransferase; ALP, alkaline phosphatase; AST, aspartate aminotransferase; BUN, blood urea nitrogen; cAMP, cyclic adenosine monophosphate; Cca/CCr, urinary calcium-to-creatinine clearance; CRP, C-reactive protein; γ-GTP, γ-glutamyl transpeptidase; HbA1c, hemoglobin A1c; HDL-C, high-density lipoprotein cholesterol; LDH, lactate dehydrogenase; LDL-C, low-density lipoprotein cholesterol; PTH, parathyroid hormone; PTHrP, PTH-related peptide; 25(OH)D₃, 25-hydroxyvitamin D₃; 1,25(OH)D₃, 1,25-dihydroxyvitamin D₃; TRP, tubular reabsorption of phosphate

Peripheral blood	Test value	Reference range
Red blood cells	431 × 10^4^/μL	420-580 ×10^4^/μL
Hemoglobin	14.3 g/dL	13.0-17.0 g/dL
Hematocrit	44.20%	38-50%
White blood cells	6,870 /μL	4,000-10,000/μL
Platelet	32.1 × 10^4^/μL	15.0-40.0×10^4^/μL
Electrolytes		
Sodium	141 mmol/L	135-145 mmol/L
Potassium	3.5 mmol/L	3.5-5.0 mmol/L
Chloride	102 mmol/L	98-108 mmol/L
Calcium	12.9 mg/dL	8.5-10.3 mg/dL
Phosphorus	2.2 mg/dL	2.5-4.5 mg/dL
Magnesium	2.5 mg/dL	1.6-2.4 mg/dL
Blood biochemistry		
Total protein	7.5 g/dL	6.5-8.0 g/dL
Albumin	4.6 g/dL	3.8-5.3 g/dL
Total bilirubin	0.8 mg/dL	0.2-1.2 mg/dL
AST	26 U/L	10-40 U/L
ALT	12 U/L	5-45 U/L
γ-GTP	27 U/L	9-35 U/L
LDH	222 U/L	120-240 U/L
ALP	113 U/L	40-130 U/L
Creatinine	0.72 mg/dL	0.6-1.1 mg/dL
BUN	13 mg/dL	8-22 mg/dL
Uric acid	5.4 mg/dL	3.6-7.0 mg/dL
CRP	0.02 mg/dL	<0.3 mg/dL
Plasma glucose	119 mg/dL	<110 mg/dL
HbA1c	5.6%	4.6-6.2%
LDL-C	87 mg/dL	<140 mg/dL
HDL-C	119 mg/dL	≥40 mg/dL
Triglyceride	48 mg/dL	<150 mg/dL
Endocrine examination		
Whole PTH	12.9 pg/mL	8.7-79.6 pg/mL
Intact PTH	172 pg/mL	10-65 pg/mL
PTHrP	<1.0 pmol/L	<1.1 pmol/L
Calcitonin	<0.5 pg/mL	<5 pg/mL
Renal cAMP	2.84 nmol/dL/GF	1.5-5.0 nmol/dL/GF
25(OH)D3	72 ng/mL	20-50 ng/mL
1,25(OH)D3	16.1 mg/dL	20-60 pg/mL
Cca/CCr ratio	0.022	<0.01-0.02
%TRP	78.22%	85-95 %

**Figure 1:**
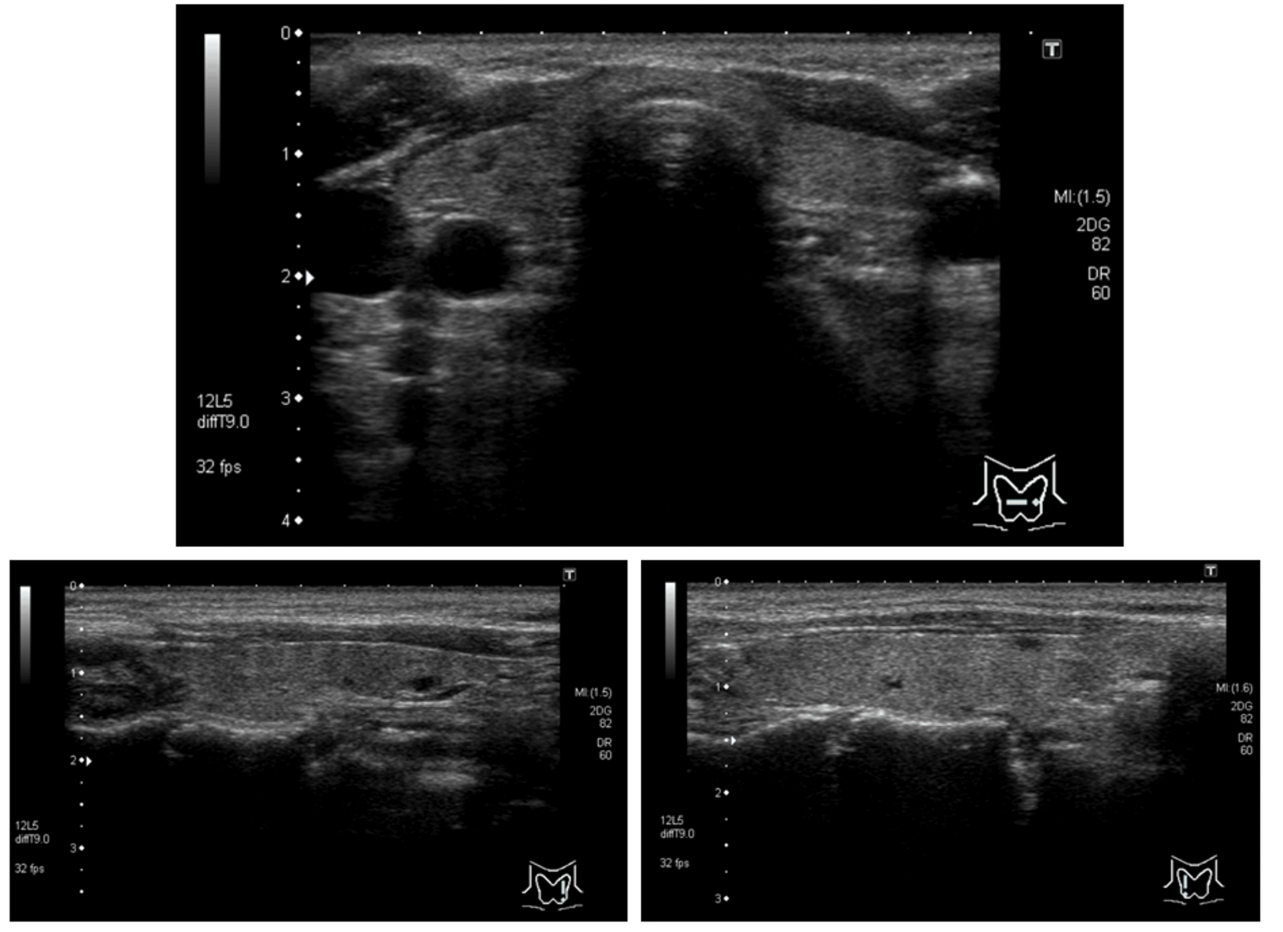
Thyroid ultrasound image No enlarged parathyroid glands were observed.

Results in urinary tests were as follows: calcium 24.9, mg/dL; urinary calcium-to-creatinine clearance (Cca/CCr) ratio, 0.022; percentage of tubular reabsorption of phosphate (TRP), 78.22. Neck ultrasonography failed to detect any enlarged parathyroid glands (Figure 1).

**Figure 2:**
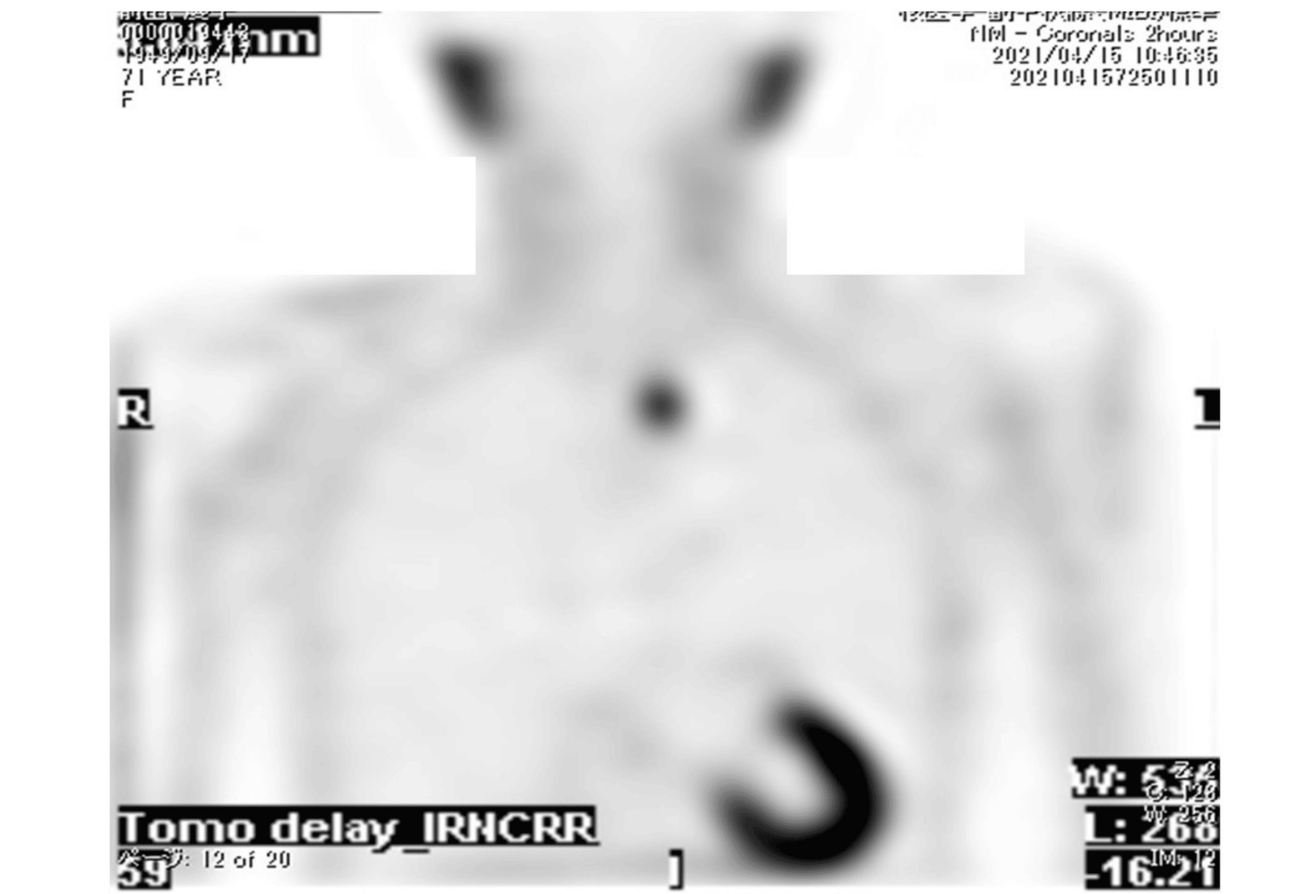
MIBI scintigraphy MIBI scintigraphy reveals a hot spot in the superior mediastinum, located at the caudal end of the left thyroid lobe, suggesting the presence of an ectopic parathyroid adenoma.

Methoxyisobutylisonitrile (MIBI) scintigraphy, however, revealed a hot spot in the superior mediastinum, located at the caudal end of the left thyroid lobe (Figure 2), suggesting the presence of an ectopic parathyroid adenoma.

**Figure 3:**
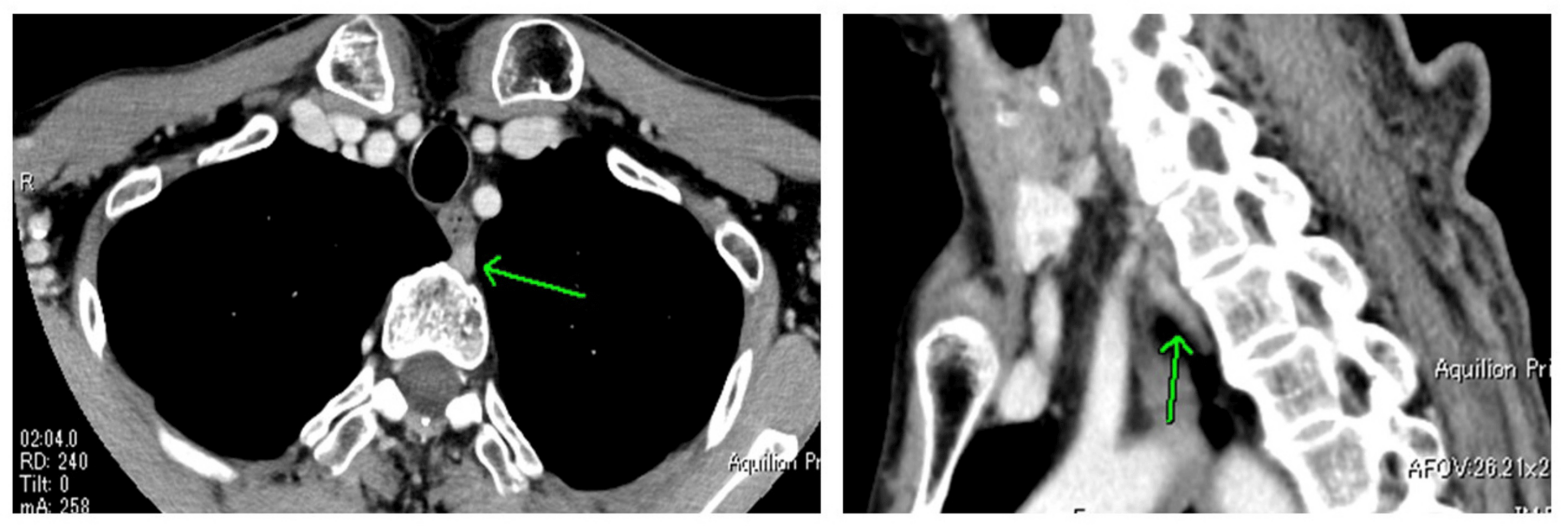
Contrast-enhanced CT Contrast-enhanced CT reveals a nodular mass with contrast enhancement between the anterior surface of the vertebral body at the Th1-2 level and the esophagus. There was no significant lymphadenopathy.

Contrast-enhanced CT imaging identified a nodular mass between the anterior vertebral body at the Th1-2 level and the esophagus, which demonstrated contrast enhancement along the craniocaudal axis. There was no significant lymphadenopathy (Figure 3).

**Figure 4:**
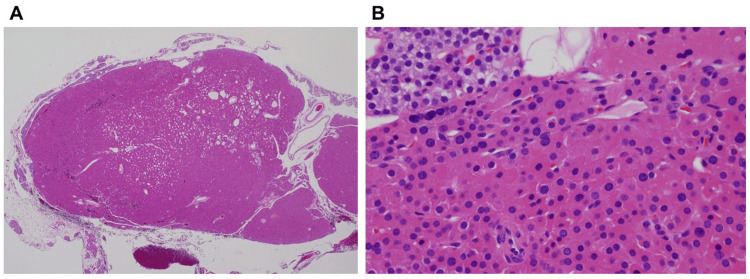
Histopathological findings (A) Magnification ×20. Parathyroid tissue, presumed to be a normal rim, is observed adjacent to the nodular lesion. (B) Magnification ×400. The nuclei of the tumor cells are slightly enlarged compared to the surrounding parathyroid tissue, suggesting the possibility of an adenoma. No clear malignant features are observed.

Left parathyroidectomy was performed. The excised parathyroid adenoma weighed 610 mg and measured 23 × 10 × 4 mm. Pathological examination confirmed an ectopic parathyroid adenoma adjacent to a normal rim of parathyroid tissue. The tumor cells showed slight nuclear enlargement compared to surrounding tissue, consistent with adenoma; no malignancy was noted (Figure 4).

After the surgery [4,5], serum whole PTH level decreased from 87.4 pg/mL to 17.9 pg/mL, and serum calcium level also decreased from 12.9 mg/dL to 9.5 mg/dL. Both serum PTH and calcium levels became within the reference range.

## Discussion

MIBI scintigraphy has proven to be the gold standard for preoperative localization of parathyroid adenomas, demonstrating a sensitivity of 70-83.6% and a specificity of 98.3% [6]. While ultrasound examination is a useful tool for detecting orthotopic parathyroid adenomas, MIBI is superior in detecting ectopic adenomas, with a sensitivity of 89% compared to that of 59% in ultrasound examination [3]. This is particularly important in cases of ectopic adenomas located in the thymus, mediastinum, and retroesophageal regions [3]. In this case, MIBI was crucial in identifying an ectopic parathyroid adenoma that was not detected on ultrasonography. It is noteworthy that sensitivity in MIBI increases with elevated PTH levels, but in cases with low PTH, MIBI may yield false negatives, which is a limitation during the process of diagnosis [3,6,7].

The clinical manifestations of PHPT, resulting from chronic hypersecretion of PTH and ensuing hypercalcemia, primarily affect bones and kidneys [8]. Without surgical intervention, patients can experience significant bone density loss within 10 years, particularly in areas with high cortical bone content such as the femoral neck and distal radius [9]. Renal complications, including nephrolithiasis and hypercalciuria, are also common in patients with PHPT [8]. In this case, PHPT had been suspected for 16 years, but due to limitations in diagnostic investigations and patient preference for observation, the conditions were exacerbated. By the time of diagnosis, bone mineral density had significantly declined. This underscores the importance of early intervention to prevent complications such as osteoporosis and kidney damage [8,10].

The challenge of diagnosing ectopic parathyroid adenomas, especially when ultrasonography fails to detect them, highlights the critical role of advanced imaging techniques such as MIBI scintigraphy. Early detection and precise localization of ectopic adenomas are vital for improving patient outcomes, as timely surgical intervention can normalize PTH and calcium levels and prevent the long-term complications of PHPT. In this case, MIBI scintigraphy played a key role in making the correct diagnosis, leading to normalization of the patient’s PTH and calcium levels after surgery [7,11].

## Conclusions

In conclusion, when PHPT is suspected based on elevated serum PTH and calcium levels, osteoporosis, and other clinical indicators, we should keep in mind the possibility of an ectopic parathyroid adenoma. If routine imaging, such as ultrasonography, fails to detect the lesion, MIBI scintigraphy should be employed for further investigation. Early and accurate detection of ectopic parathyroid adenomas is crucial for accurate diagnosis and improved patient outcomes, as timely surgical intervention can prevent complications and normalize serum PTH and calcium levels. While MIBI scintigraphy proved effective in localizing the ectopic parathyroid adenoma in this case, it is important to consider differential diagnoses, including thyroid neoplasms, malignancies, and lymphadenopathies. Diagnostic limitations such as false-negative MIBI results, particularly in patients with low PTH levels, should be acknowledged. In challenging cases, additional imaging modalities, such as single-photon emission computed tomography (SPECT)-CT or 4D-CT, can provide valuable complementary information to improve localization accuracy. Therefore, a comprehensive diagnostic approach combining multiple imaging techniques and thorough clinical evaluation is important to enhance diagnostic accuracy and avoid potential pitfalls.
